# A survey of lip lesions diagnosed in a single institution: A clinicopathological study

**DOI:** 10.4317/jced.62203

**Published:** 2024-12-01

**Authors:** Mayara Larissa Moura de Souza, Danielle Machado Farias, Anne Evelyn de Oliveira Moura, Jurema Freire Lisboa de Castro, Elaine Judite de Amorim Carvalho, Danyel Elias da Cruz Perez

**Affiliations:** 1 School of Dentistry, Department of Clinical and Preventive Dentistry, Oral Pathology Unit, Universidade Federal de Pernambuco, Recife, Pernambuco, Brazil; 2 Piracicaba Dental School, Oral Pathology and Semiology Area, State University of Campinas (UNICAMP), Piracicaba, São Paulo, Brazil

## Abstract

**Background:**

The aim of this study was to assess the clinicopathological features of lip lesions diagnosed in a single Oral Pathology service in Brazil.

**Material and Methods:**

It was a cross-sectional study based on secondary data. Between 2000 and 2019, all lip lesions diagnosed in an Oral Pathology service in Brazil were analyzed. Clinical and demographic data, such as patient age and gender, general habits, location, clinical hypotheses of diagnosis, and biopsy type, were collected from patients’ clinical records. All cases were microscopically reviewed. Descriptive statistics were obtained for all described variables. Subsequently, associations between variables and identified lesion groups were performed. The KAPPA test was used to assess the agreement between clinical and histopathological diagnoses.

**Results:**

A total of 1,284 cases were analyzed, representing 17.8% of oral lesions. The most prevalent lesions were: mucocele (32.9%), fibrous hyperplasia (15.7%), non-specific chronic sialadenitis (11.1%), and actinic cheilitis (9.6%). The demographic distribution included 740 women (61%) and 472 men (39%). The mean age was 37.6 years (ranging from 3 to 97 years). The primary site of presentation was the lower lip, with 980 cases (86.7%). In 783 cases (64.5%), there was concordance between the clinical and histopathological diagnoses.

**Conclusions:**

Lip is a significant region for oral diseases, representing approximately 20% of all oral lesions. Importantly, more than 10% of the cases were oral potentially malignant disorders or malignant neoplasms.

** Key words:**Lip, Lip diseases, Epidemiology.

## Introduction

The lips constitute the primary barrier of the oral cavity and, owing to their mobility and presence of several different tissues, are regarded as anatomical structures of significant interest in the study of the facial complex. For these reasons, the lips present potential to exhibit a spectrum of lesions from distinct origin, ranging from traumatic, infectious, and inflammatory conditions to benign and malignant neoplasms (1). Some dermatological and systemic diseases may exhibit labial manifestations, such as systemic lupus erythematosus, multiform erythema, and Sjögren’s Syndrome. Therefore, the lips constitute an important anatomical region to be meticulously examined through rigorous inspection and palpation. If indicated, biopsy and subsequent histopathological analysis are mandatory for the diagnosis of both, local and systemic diseases (2).

Some studies on the prevalence and epidemiology of labial lesions have been conducted. Lip lesions represent around 15% of all oral diseases. The sample size and the prevalence of the lesions vary in each study (1,3). Hence, it is imperative for the practitioners, especially dentists, to have appropriate training to enable correct diagnosis and treatment, given the common occurrence of lip lesions in routine practice. Recognition of these alterations, meticulous recording of medical history and current illness, as well as thorough oral clinical examination, contribute to the identification and diagnosis of the lesions. Few studies have evaluated and discussed exclusively lip diseases (1-3,5). Thus, the aim of this study was to assess the clinicopathological features of lip lesions diagnosed in a single Oral Pathology service in Brazil.

## Material and Methods

This was a cross-sectional study based on secondary data. The study was approved by the local Institutional Research Board (protocol # 46177521.0.0000.5208). Between 2000 and 2019, all lip lesions diagnosed in the Oral Pathology service, Federal University of Pernambuco, Recife, Brazil, were analyzed. Clinical and demographic data, such as patient age and gender, general habits, location, clinical hypotheses of diagnosis, and biopsy type, were collected from patients’ clinical records. All cases were microscopically reviewed.

The analysis of clinical records was performed by researchers with expertise in the diagnosis of oral lesions. The sample included patients with lesions located on the upper and lower lips (mucosa and/or semi-mucosa), of all ages, and only those who underwent biopsy for diagnostic purposes were considered. Cases without any clinical information and those lacking records of histopathological diagnosis, as well as those without adequate material for histopathological review, were excluded from the analysis.

The patients were categorized into age groups according to the WHO criteria: child (0-9 years), young (10-19 years), adult (20-59 years), and elderly (60 years or older) (4). According to the origin of the lesion, the diseases were grouped into normal tissue, normal variations of soft tissue, non-odontogenic cysts, pigmented lesions, infectious diseases, immunologically mediated diseases, non-neoplastic salivary gland diseases, reactive/inflammatory lesions, oral potentially malignant diseases, benign neoplasms, and malignant neoplasms.

According to the location, cases were grouped as follows: upper lip, lower lip, both upper and lower lips simultaneously, and multiple lesions (when they affect other oral regions besides the lips). The type of biopsy was also categorized as incisional and excisional. The correlation between clinical and microscopic diagnosis was analyzed by comparing all clinical hypotheses with the histopathological diagnosis. If there was agreement with any of the hypotheses, it was considered that the clinical diagnosis was confirmed by histopathological analysis.

The collected data were entered and stored in a Microsoft Excel spreadsheet, which was exported and analyzed using the IBM® Statistical Package for the Social Sciences® software (IBM SPSS Statistics for Windows, Version 24.0. Armonk, NY: IBM Corp). Descriptive statistics were applied to all variables described by the calculation of absolute and relative frequencies. Subsequently, associations between variables and each lesion group were examined. The KAPPA test was conducted to determine the degree of agreement between clinical and histopathological diagnoses for each lesion.

## Results

From a total of 7182 lesions, 1,357 (18.9%) were located in the lips. Seventy-three cases were excluded due to a lack of clinical information and/or adequate material for histopathological review. Thus, 1,284 cases (17.8%) were included in the study. The cases were grouped according to the nature of lesions, and the most frequent lesions are shown in  Table 1.

Most cases occurred in the lower lips (980 – 86.7%) and in women (740 - 61%). Only 2 cases occurred on both lips, comprising 1 case of squamous papilloma and 1 case of melanocytic macule, while 16 were considered multiple lesions due to involvement of other regions of the oral cavity besides the lips.

The mean age was 37.6 years (ranging from 3 to 97 years). The adults (20-59 years) were the most affected, comprising 591 (53.6%) lesions, followed by young (204, 18.5%) elderly individuals (191, 17.3%), and children (115, 10.4%). The age information was missing in 183 cases ( Table 2).

The most frequent type of biopsy was excisional, accounting for 663 cases (70.3%). Histopathological analysis confirmed the clinical diagnosis in 783 cases (64.5%). The group of immunologically mediated lesions showed the highest agreement, with a KAPPA value of 0.875. No agreement between clinical and histopathological diagnosis was observed in 9 (24.3%) cases of malignant neoplasms.

The associations between variables based on each lesion group and age group are depicted in  Table 2. Normal variations of soft tissue, pigmented lesions, immunologically mediated lesions, non-neoplastic salivary gland disease and reactive/inflammatory lesions were more observed in females, while infectious diseases, benign neoplasms, oral potentially malignant disorders and malignant neoplasms were more frequent among males. For non-odontogenic cysts, there was not sex predilection ( Table 2). The most prevalent age group for all lesion groups was adults, except for non-odontogenic cysts. Only non-odontogenic cysts and benign neoplasms were more common in upper lips.

In normal variations of soft tissue (32 - 100%), venous malformations comprised 17 cases (53.1%). In the pigmented lesions (30 - 100%), 26 cases (86.7%) were diagnosed as melanocytic macule. Only 3 (10%) cases were melanocytic nevus and 1 case (3.3%) was diagnosed as blue nevus. In the infectious lesions (6 - 100%), there were 2 cases of paracoccidioidomycosis, 2 of verruca vulgaris, and 2 of condyloma acuminatum. Regarding to the immunologically mediated lesions (32 - 100%), 19 cases (59.4%) were recorded as focal chronic sialoadenitis (spectrum of the Sjogren syndrome), 7 cases (21.9%) were vulgar pemphigus, 3 (9.4%) erythema multiforme, 2 (6.2%) mucous membrane pemphigoid, and 1 case (3.1%) of lupus erythematosus.

There were 422 (73.6%) cases of mucocele, representing the most common non-neoplastic salivary gland disease (574 - 100%), followed by non-specific chronic sialadenitis (142, 24.8%), salivary duct cyst (6, 1%), sialolithiasis (3, 0.5%), and 1 case (0.1%) of glandular hyperplasia. In reactive/inflammatory lesions (309 - 100%), 202 cases (65.4%) corresponded to fibrous hyperplasia, 44 cases (14.2%) were pyogenic granuloma, 44 (14.2%) non-specific chronic inflammatory process, 9 (3%) epithelial hyperplasia, 6 (2%) non-specific ulcer, 2 (0.6%) granulation tissue and 2 cases (0.6%) of lichenoid reaction. Regarding to the benign neoplasms (65 - 100%), there were 33 cases (51%) of squamous papilloma, 12 (18.5%) pleomorphic adenoma, 9 (13.8%) lipomas, 5 (7.6%) neuromas, 4 (6.1%) canalicular adenomas and 2 (3%) neurofibromas ( Table 3).

All cases of oral potentially malignant disorders were actinic cheilitis (124 - 100%). There were 29 (78.3%) cases of squamous cell carcinoma, the most common malignant neoplasm (37 - 100%), followed by 3 cases (8.1%) of basal cell carcinoma. One case each of mucoepidermoid carcinoma, adenoid cystic carcinoma, polymorphous adenocarcinoma, leiomyosarcoma, and metastatic breast carcinoma were also observed ( Table 4).

## Discussion

In this survey, 17.8% of oral diseases were located in lips, mainly in adults between 20-59 years. Other series found that the lip lesions represented between 10% and 16% of all oral diseases (3,5). Females were more affected (3). However, a male predilection has been also recorded (5). The sample size and differences associated with the geographic region, considering distinct habits and culture, may explain these different findings regarding to the sex prevalence in lip lesions.

Lower lip was the most common site for all group of lesions, except for benign salivary gland tumors and non-odontogenic cysts as observed in other series (3,6). Palate is the most common site for oral pleomorphic adenoma, followed by upper lip. Canalicular adenoma affects more commonly the upper lip (6,7). Thus, the site is a relevant aspect for the diagnosis of labial submucous nodule. Benign salivary gland neoplasms, particularly pleomorphic adenoma and canalicular adenoma, constitute the main differential diagnoses for submucosal nodules located in the upper lip (1,6,7).

The most prevalent histopathological diagnosis was mucocele, with a predominance in the lower lip. This can be explained by the anatomical position of the lower lip, which is more predisposed to traumas. Fibrous hyperplasia, the second most frequent lesion, represents a reactive/inflammatory lesion also caused by an irritative factor, such as chronic trauma. This lesion was also found more frequent in the lower lip, likely due to a higher chance of traumas in that region (3).

The group of immunologically mediated lesions showed the highest prevalence in females. Most cases included in this group were diagnosed as focal chronic sialoadenitis, which is an important histopathological finding for the diagnosis of Sjögren’s syndrome (8,9). In the current study, all cases of focal chronic sialoadenitis were consistent with the histological spectrum of Sjogren syndrome, with lymphocytic foci adjacent to mucous acini and ducts, containing more than 50 lymphocytes per mm2 of glandular tissue (8).

Despite being endemic in the southeast and central-west regions of Brazil, the paracoccidioidomycosis has low incidence in the northern and northeastern regions of the country, with the exception of the states of Pará and Rondônia (10). This likely explains the low prevalence of paracoccidioidomycosis observed in this sample, from the northeastern region of Brazil. In this series, the 2 cases of paracoccidioidomycosis occurred in multiple sites of oral mucosa, affecting the lower lip of men. The disease has a strong preference for middle-aged men, usually residing in rural areas. Women are less affected due to the female hormone estradiol 17-β, which inhibits the transition from mycelium or conidium to yeast (pathogenic form), preventing the progression of the disease (10).

Actinic cheilitis, an oral potentially malignant disorder, represented almost 10% of the sample. A study found a prevalence of 23% (3). Overall, the malignant transformation rate of actinic cheilitis is 10%. Clinically, the lesion appears as a white plaque, with atrophic/erythematous areas. Ulcers may also be observed. Biopsy is essential to evaluate the lip epithelium, with subsequent guidance and follow-up of the patient. Most patients are male adults (3,11), as observed in this study. In the same way, all cases of squamous cell carcinoma occurred in the lower lip, most of them in men. Brazil is a tropical country with high levels of UV radiation. In this study, conducted in an institution located in a coastal city in the Northeast region of Brazil, there are many rural workers, street vendors and fishermen who are exposed to high levels of UV radiation daily. This fact explains the high prevalence of actinic cheilitis and squamous cell carcinoma, and the need to massively educate people about taking protective measures against UV radiation. However, it should be highlighted that 30% of squamous cell carcinoma affected women, emphasizing the need to pay attention to clinical signs of malignancy in women as well, such as ulcerated lesions with indurated borders or erythematous macules of unknown origin. In these cases, biopsy is mandatory. Regarding the 3 cases of basal cell carcinoma, the tumors involved the lip semi-mucosa and skin.

The concordance rate between clinical and histopathological diagnosis was observed in 64.5% of the cases. The groups with the lowest concordance rates were non-odontogenic cysts (25%) and normal variations of soft tissue (28.1%). These data may reveal the inability of some dentists in diagnosing oral lesions, especially normal variations of soft tissue, such as Fordyce granules, where biopsy is unnecessary. On the other hand, all other groups achieved a diagnostic concordance rate above 59%, with the group of immunologically mediated lesions showing the highest rate. Particularly in this group, most patients presented clinical signs and symptoms of Sjogren syndrome. Thus, the lip biopsy was performed to evaluate the minor salivary glands as criteria for diagnosis. Moreover, although a substantial concordance rate was observed within the malignant neoplasms group, no agreement was observed in 24.3% of the malignant tumors. This data is relevant, considering the importance of proper evaluation and approach for the diagnosis and prognosis of these lesions.

A limitation of this study was the inclusion of lesions diagnosed solely through anatomopathological examination. Thus, common oral lesions, such as oral herpes simplex and angular cheilitis were not addressed, because their diagnosis is established almost exclusively based on clinical appearance and anamnesis.

In conclusion, the lips represent almost 20% of all oral diseases. Although most of them are non-neoplastic, malignant neoplasms or oral potentially malignant disorders present significant prevalence. In addition, salivary gland tumors should be considered in the differential diagnosis of submucous nodule in the upper lip.

## Figures and Tables

**Table 1:** Main histopathological diagnoses of lip lesions.

Histopathological diagnosis*	Frequency
Mucocele	422 (32.9%)
Fibrous hyperplasia	202 (15.7%)
Non-specific chronic sialadenitis	142 (11.1%)
Actinic cheilitis	124 (9.6%)
Normal tissue	71 (5.5%)
Pyogenic granuloma	44 (3.4%)
Non-specific inflammatory process	44 (3.4%)
Squamous papiloma	33 (2.6%)
Squamous cell carcinoma	29 (2.3%)
Melanocytic macule	26 (2.0%)
Focal chronic sialoadenitis (spectrum of Sjögren's syndrome)	19 (1.5%)
Other	128 (10%)
Total	1,284 (100%)

*Other diagnoses represented an insignificant percentage for each disease

**Table 2:** Distribution of cases and association of lesion groups with sex and age, by age group according to WHO criteria.

Lesion group	Sex		Age Group			
	Male	Female	Child	Young	Adult	Elderly
Normal variations of soft tissue^1^	11 (34.4%)	21 (65.5%)	0 (0.0)	1 (3.1%)	21 (65.6%)	8 (25%)
Non-odontogenic cysts^2^	2 (50%)	2 (50%)	0 (0.0)	1 (25%)	1 (25%)	1 (25%)
Pigmented lesions^3^	8 (26.7%)	22 (73.3%)	0 (0.0)	2 (6.7%)	17 (56.7%)	8 (26.7%)
Infectious diseases	4 (66.7%)	2 (33.3%)	0 (0.0)	1 (16.7%)	3 (50%)	2 (33.3%)
Immunologically mediated diseases	8 (25%)	24 (75%)	1 (3.1%)	0 (0.0)	26 (81.3%)	5 (15.6%)
Non-neoplastic salivary gland disease^4^	204 (35.5%)	370 (64.5%)	96 (16.7%)	149 (26%)	233(40.6%)	42 (7.3%)
Reactive/inflammatory lesions^5^	98 (31.7%)	210 (68%)	9 (2.9%)	35(11.3%)	185(59.9%)	51(16.5%)
Benign neoplasms^6^	35 (53.8%)	30 (46.2%)	7 (10.8%)	13 (20%)	23 (35.4%)	12(18.5%)
Oral potentially malignant disorders^7^	76 (61.3%)	48 (38.7%)	1 (0.8%)	2 (1.6%)	65 (52.4%)	46(37.1%)
Malignant neoplasms8	26 (70.3%)	11 (29.7%)	1 (2.7%)	0 (0.0)	17 (45.9%)	16(43.2%)
Total^*^	472 (39%)	740 (61%)	115 (10.4%)	204 (18.5%)	591 (53.7%)	191 (17.4%)

1Two cases did not report age; 2One case did not report age; 3Three cases did not report age; 4Four cases did not report age; 5One case did not report sex; 610 cases did not report age; 710 cases did not report age; 8Eight cases did not report sex; *71 cases of normal tissue were not included.

**Table 3:** Distribution of benign neoplasms according to sex, location, and age.

Benign neoplasms	Sex		Location			Mean age (Years, range)
	Male	Female	Upper	Lower	Upper and lower	
Pleomorphic adenoma^*^	7 (58.4%)	5 (41.6%)	11 100%)	0	0	32.6 (18-65)
Canalicular adenoma	3 (75%)	1 (25%)	4 (100%)	0	0	49.5 (18-81)
Lipoma	7 (77.8%)	2 (22.2%)	1 (11.1%)	8 (88.8%)	0	59 (21-97)
Papilloma^±^	15 (45.5%)	18 (54.5%)	12(42.8%)	15 (53.6%)	1(3.6%)	29.1 (6-75)
Neuroma	3 (60%)	2 (40%)	1 (20%)	4 (80%)	0	34.4 (6-68)
Neurofibroma	0	2 (100%)	1 (50%)	1 (50%)	0	78 (66-90)
Total	35 (53.8%)	30 (46.1%)	30(50.8%)	28 (47.4%)	1(1.6%)	

*In 1 case, the site was not available ±In 5 cases, the site was not available

**Table 4:** Distribution of histopathological diagnoses of the malignant neoplasms according to sex, location, and age.

Malignant neoplasms	Sex		Location			Mean age (Years, range)
	Male	Female	Upper	Lower	Multiple lesions	
Squamous cell carcinoma^*^	21(72.4%)	8 (27.6%)	1 (3.6%)	26 (92.8%)	1 (3.6%)	61.8 (32-86)
Basal cell carcinoma	3 (100%)	0	2 (66.7%)	1 (33.3%)	0	44.5 (29-60)
Mucoepidermoid carcinoma	1 (100%)	0	0	1 (100%)	0	53
Adenoid cystic carcinoma	0	1 (100%)	0	1 (100%)	0	NA**
Polymorphous adenocarcinoma	0	1 (100%)	1 (100%)	0	0	30
Leiomyosarcoma	1 (100%)	0	0	1 (100%)	0	5
Metastatic breast carcinoma	0	1 (100%)	1 (100%)	0	0	80
Total	26 (70.3%)	11 (29.7%)	5 (13.9%)	30 (83.3%)	1 (2.8%)	

*In one case, the site was not available **Not available

## Data Availability

The databases used and analyzed during the study are available from the corresponding author.

## References

[1] Kalogirou EM, Balta MG, Koufatzidou M, Tosiou A, Tosios KI, Nikitakis NG (2021). Tumors of the labial mucosa: a retrospective study of 1045 biopsies. Med Oral Patol Oral Cir Bucal.

[2] Greenberg SA, Schlosser BJ, Mirowski GW (2017). Diseases of the lips. Clin Dermatol.

[3] Barros CC, Medeiros CK, Rolim LS, Cavalcante IL, Santos PP, Silveira ÉJ (2020). A retrospective 11-year study on lip lesions attended at an oral diagnostic service. Med Oral Patol Oral Cir Bucal.

[4] Baer B, Bhushan A, Taleb HA, Vasquez J, Thomas R (2016). The right to health of older people. Gerontologist.

[5] Bansal S, Shaikh S, Desai RS, Ahmad I, Puri P, Prasad P (2017). Spectrum of lip lesions in a tertiary care hospital: an epidemiological study of 3009 Indian patients. Indian Dermatol Online J.

[6] Kokubun K, Chujo T, Yamamoto K, Akashi Y, Nakajima K, Takano M (2023). Intraoral minor salivary gland tumors: a retrospective, clinicopathologic, single-center study of 432 cases in Japan and a comparison with epidemiological data. Head Neck Pathol.

[7] Pires FR, Pringle GA, de Almeida OP, Chen SY (2007). Intra-oral minor salivary gland tumors: a clinicopathological study of 546 cases. Oral Oncol.

[8] Bautista-Vargas M, Vivas AJ, Tobón GJ (2020). Minor salivary gland biopsy: Its role in the classification and prognosis of Sjögren's syndrome. Autoimmun Rev.

[9] Ayesha B, Fernandez-Ruiz R, Shrock D, Snyder BM, Lieberman SM, Tuetken R (2021). Clinical and laboratory features of patients with focal lymphocytic sialadenitis on minor salivary gland biopsy for sicca symptoms: A single-center experience. Medicine (Baltimore).

[10] Souza RL, Bonan PR, Pinto MB, Prado JD, de Castro JF, Carvalho EA (2019). Oral paracoccidioidomycosis in a non-endemic region from Brazil: A short case series. J Clin Exp Dent.

[11] Rodríguez-Blanco I, Flórez Á, Paredes-Suárez C, Rodríguez-Lojo R, González-Vilas D, Ramírez-Santos A (2019). Actinic cheilitis: analysis of clinical subtypes, risk factors and associated signs of actinic damage. Acta Derm Venereol.

